# The Pragmatic Classification of Upper Extremity Motion in Neurological Patients: A Primer

**DOI:** 10.3389/fneur.2019.00996

**Published:** 2019-09-18

**Authors:** Avinash Parnandi, Jasim Uddin, Dawn M. Nilsen, Heidi M. Schambra

**Affiliations:** ^1^Department of Neurology, New York University School of Medicine, New York, NY, United States; ^2^Department of Neurology, Columbia University Medical Center, New York, NY, United States; ^3^Department of Rehabilitation and Regenerative Medicine, Columbia University Medical Center, New York, NY, United States; ^4^Department of Rehabilitation Medicine, New York University School of Medicine, New York, NY, United States

**Keywords:** machine learning algorithms, wearable sensors, inertial measurement unit, accelerometers, functional primitives, stroke rehabilitation

## Abstract

Recent advances in wearable sensor technology and machine learning (ML) have allowed for the seamless and objective study of human motion in clinical applications, including Parkinson's disease, and stroke. Using ML to identify salient patterns in sensor data has the potential for widespread application in neurological disorders, so understanding how to develop this approach for one's area of inquiry is vital. We previously proposed an approach that combined wearable inertial measurement units (IMUs) and ML to classify motions made by stroke patients. However, our approach had computational and practical limitations. We address these limitations here in the form of a primer, presenting how to optimize a sensor-ML approach for clinical implementation. First, we demonstrate how to identify the ML algorithm that maximizes classification performance and pragmatic implementation. Second, we demonstrate how to identify the motion capture approach that maximizes classification performance but reduces cost. We used previously collected motion data from chronic stroke patients wearing off-the-shelf IMUs during a rehabilitation-like activity. To identify the optimal ML algorithm, we compared the classification performance, computational complexity, and tuning requirements of four off-the-shelf algorithms. To identify the optimal motion capture approach, we compared the classification performance of various sensor configurations (number and location on the body) and sensor type (IMUs vs. accelerometers). Of the algorithms tested, linear discriminant analysis had the highest classification performance, low computational complexity, and modest tuning requirements. Of the sensor configurations tested, seven sensors on the paretic arm and trunk led to the highest classification performance, and IMUs outperformed accelerometers. Overall, we present a refined sensor-ML approach that maximizes both classification performance and pragmatic implementation. In addition, with this primer, we showcase important considerations for appraising off-the-shelf algorithms and sensors for quantitative motion assessment.

## Introduction

Wearable sensors, such as inertial measurement units (IMUs) and accelerometers, provide an opportunity for the objective, and seamless capture of human motion. Machine learning (ML) enables computers to learn without being explicitly programmed, and provides an opportunity to rapidly identify patterns in data. ML is potentially a powerful tool for clinical application because of its ability to automatically recognize categories of interest. These categories could be used for diagnostic purposes (e.g., severity of disease, disease identification) or therapeutic purposes (e.g., dose quantitation during stroke rehabilitation).

Given recent technological and computational advances, combining wearable sensor data with ML algorithms has the potential for rapid, automated, and accurate classification of motion. Researchers have begun using this combined sensor-ML approach in a number of applications. These include human activity recognition (1–3), gesture analysis (4), assessment of bradykinesia in Parkinson's disease (5, 6), motor function assessment in multiple sclerosis (7), and differentiating between functional and non-functional arm usage in stroke patients (8, 9). While many of these studies showcase the application of sensors and ML in clinical populations, no previous work has detailed the various hardware and software considerations for using the sensor-ML approach. Furthermore, no guide currently exists to advise investigators in building and troubleshooting this approach, which sits at the intersection of human movement science, data science, and neurology. With the potential for the sensor-ML approach to have widespread applicability to neurological disorders, understanding how to develop this approach for one's own area of inquiry is paramount.

One possible application of the combined sensor-ML approach is the monitoring of rehabilitation dose in stroke patients. Quantifying the dose of rehabilitation entails classifying units of measurement, which are subsequently tallied. In our previous proof-of-principle study, we used IMUs worn by stroke subjects performing a structured tabletop activity to capture motion data. Our units of measurement were functional primitives, elemental motions that cannot be further decomposed by a human observer. We applied an ML algorithm (hidden Markov model with logistic regression) to the IMU motion data to recognize primitives embedded in this activity, achieving an overall classification performance of 79% (10). While promising, this sensor-ML approach had variable classification performance among the primitives (62–87% accuracy). It also did not address research implementation challenges such as the computational complexity and computational costs of the ML approach, or clinical implementation challenges such as the expense (11) and electromagnetic intolerance of the IMUs.

In the present study, we address these limitations in the form of a primer, outlining deliberations that researchers developing their own sensor-ML approach would need to consider. We describe our rationale and steps for identifying (1) an algorithm that is highly accurate but computationally tractable, and (2) the type and array of sensors that minimize cost but maximize accuracy. We use functional primitives as the motion type to be classified, and describe our approach for both capturing and identifying these motions. We also use off-the-shelf algorithms and sensors, providing an accessible framework for investigators seeking to address new scientific and clinical questions with the sensor-ML approach.

## Methods

**Figure 1:**
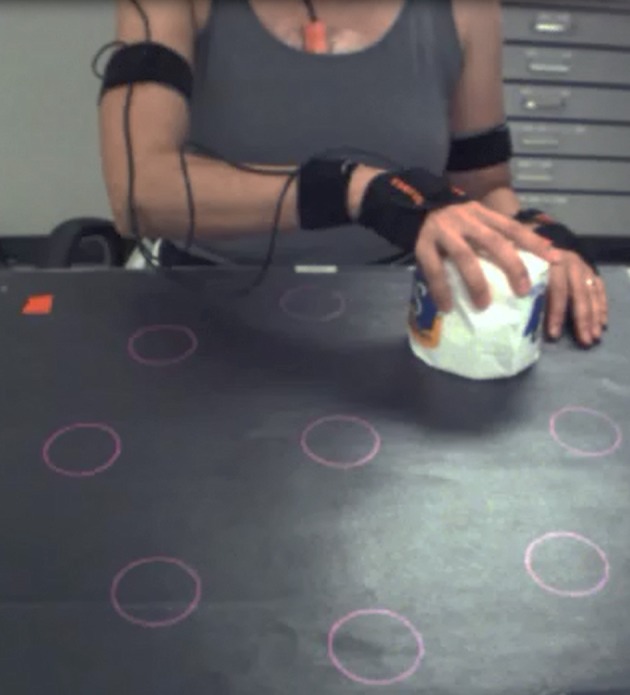
Tabletop activity set-up. Healthy individual wearing the sensors and transporting the object from center to a target.

To demonstrate the steps in identifying the optimal ML algorithm and sensor array, we use data collected from previous work (10). Briefly, six mild-to-moderately impaired stroke patients (Table 1) moved a toilet paper roll and aluminum can over a horizontal array of targets (Figure 1).

**Table 1:** Demographic and clinical characteristics of patients.

*N*	6
Age (years)	61.7 (46.5–71.0)
Gender (Female/Male)	2F/4M
Dominant arm (Right/Left)	5R/1L
Paretic side (Right/Left)	6R
Impairment (Fugl-Meyer score)	52.8 (45–62)
Time since stroke (years)	12.0 (2.0–31.1)

*Shown are number of participants, mean age (range), gender, race, hand dominance, paretic side, mean Fugl-Meyer assessment score at first assessment (range; maximum 66), and time since stroke (range). Inclusion criteria were age ≥18 years; premorbid right-hand dominance; unilateral motor stroke; contralateral arm weakness with Medical Research Council score <5/5 in a major muscle group. Exclusion criteria were traumatic brain injury; musculoskeletal, medical, or non-stroke neurological condition interfering with assessment of motor function; contracture at shoulder, elbow, or wrist; moderate dysmetria or truncal ataxia; visuospatial neglect; apraxia; global inattention; blindness*.

Subjects performed 5 trials moving the object between a center target and eight radially arrayed targets (20 cm away). The task generates the following functional primitives: *reach* (to move into contact with a target object); *transport* (to convey a target object); *reposition* (to move proximate to a target object); and *idle* (to stand at the ready near target object). Functional primitives are discrete, object-oriented motions with a single goal (12). Functional primitives are non-divisible and are largely invariant across individuals (13), may be represented cortically (14–16), and provide a finer-grained capture of performance in stroke patients who may be unable to accomplish a full activity. Akin to words, functional primitives are combined to make a functional movement (17) (analogous to a sentence), which in turn are combined to make an activity (analogous to a paragraph) (18). For example, a series of *reach-transport-reposition* primitives could constitute a functional movement for zipping up a jacket, within the activity of dressing.

Motion data were recorded with 11 IMUs (XSens Technology) worn on the head, sternum, pelvis, and bilateral hands, forearms, arms, and scapulae. 3D linear accelerations, 3D angular velocities, and quaternions were generated at 240 Hz. To segment and label the motion data as constituent functional primitives, we synchronously recorded motion (30 Hz) with a single video camera. Trained coders used the video recording to label the beginning and end of each primitive, which also labeled the corresponding IMU data. These labels served as the ground truth. This step enabled us to train ML algorithms on motion data and test their classification performance against the ground-truth labels. IMU data were z-score normalized and statistical features were extracted. Following prior work, the statistical features were the following: mean, standard deviation, minimum, maximum, and root mean square (10). These statistical features have been shown to capture human motion efficiently, reducing the computational burden (19–21). We selected a window size of 0.25 s sliding by 0.1 s (10), from which to derive the statistical features. The statistical feature data were fed to the ML algorithms.

The dataset consisted of 2,881 functional primitives, consisting of 810 *reaches*, 708 *transports*, 781 *repositions*, and 582 *idles*. It is important to note that this is the sample size of interest (not the number of subjects). Accounting for repeated measures within-subject and at each target, and using this dataset of 2,881 primitives with α = 0.05, we have 81% power to detect a classification performance of at least 79% (positive predictive value, section Classification Performance of Algorithms below). We used 79% accuracy as the benchmark for sufficient classification performance as achieved in our previous study (10).

## Computational Details

### ML Methods for Classification

In the present study, we sought to identify an ML algorithm that performs well for identifying functional primitives, i.e., has a high classification performance, but that also is practical, i.e., has low computational overhead and minimal tuning requirements. Supervised ML algorithms work in two phases: training and testing. During training, ML algorithms learn the relationship between a pattern of data characteristics (here, the statistical features) and its class (here, its primitive label). During testing, the trained ML algorithm uses the pattern of data characteristics to identify a new data sample as one of the primitives. This identification is checked against the ground-truth human label, thus reading out classification performance.

We considered both generative and discriminative algorithms. Generative algorithms model the underlying distribution of data for each class, seeking to identify data characteristics that enable matching of new data samples to a given class. In contrast, discriminative algorithms model the boundaries between classes and not the data themselves. They seek to identify the plane separating the classes so that, based on location relative to the plane, a new data sample is assigned to the appropriate class.

We selected four algorithms that have been found to provide high classification performance in human activity recognition: linear discriminant analysis (LDA) (22), Naïve Bayes classifier (NBC) (19), support vector machine (SVM) with a radial basis function kernel (23), and k-nearest neighbors (KNN) (20). LDA and NBC are generative algorithms, whereas SVM and KNN are discriminative algorithms. We used off-the-shelf versions of these algorithms without any special permutations; in other words, the algorithms are widely available in most machine learning libraries such as scikit-learn (24, 25).

### Algorithm Performance Metrics

#### Classification Performance of Algorithms

We first evaluated how well the algorithms could classify primitives, measuring classification performance by comparing algorithm-chosen labels against ground-truth human labels. Primitives were classified as true positive (*TP*, labels agreed) and false positive (*FP*, labels disagreed). We used 60% of the data to train the algorithm and 40% to test it, repeating the process 10 times. A validation dataset was not used because we were not optimizing algorithm architectures, and the test dataset provides an unbiased estimate of algorithm performance. Data were randomly selected for each primitive proportional to its prevalence in the complete dataset (i.e., stratified proportional sampling). This ensured that each dataset adequately represented the entire sample population. In addition, to examine the possibility that within-subject dependencies in the training and testing sets leads to an overestimation of classification performance, we also performed a leave-one-subject-out analysis i.e., training the algorithms using data from all but one subject and testing its performance on the data from the remaining subject. This process was repeated 6 times, once for each subject, and classification performances were averaged.

The first metric for classification performance was positive predictive value (PPV; *[TP/(TP*+FP)]^*^100). PPV reflects how often a primitive was actually performed when the algorithm labeled it as such; in other words, PPV is how often a primitive was correctly classified. We generated primitive-level PPVs in a one-vs.-all analysis (e.g., *reach* vs. *transport* + *reposition* + *idle* combined). We also generated an overall PPV by combining data for all primitives and tallying all true and false positives. We prefer PPV because it takes into account the prevalence of the primitive in the dataset (26). We additionally examined confusion matrices for each algorithm. A confusion matrix allows us to visualize where the algorithm is succeeding or failing in its classification. Each row of the confusion matrix correspond to the true classes (human-generated) and each column correspond to the predicted-classes (ML-generated). The diagonal cells between the rows and the columns represent the percentage of primitives for which the predicted class is same as the true class, while the adjacent cells (non-diagonal) represent the percentage of primitives misclassified by the algorithm. Ideally, the diagonal cells should contain a value of 100% with the adjacent cells being 0%, indicating perfect classification.

The second metric for classification performance was the receiver operating characteristic (ROC) curve. ROC curves depict the relative tradeoff between true positive rate (sensitivity; y-axis) and false positive rate (1-specificity; x-axis) and identify the optimal operating point of an algorithm (27). Perfect classification would lead to a ROC curve that passes through the upper left corner, with an area under the ROC curve (AUC) equal to 1 and an operating point at 100% sensitivity and 100% specificity (27).

#### Practical Performance of Algorithms

We next considered the computational complexity of the algorithms in terms of their training and testing times and their tuning requirements. Having a high computational complexity means that specialized computing hardware and advanced expertise would be needed, potentially hindering widespread implementation in research.

##### Training and testing times of the algorithms

The time required to train and test the algorithms was measured for datasets of different sizes. If training time is fast, rapid appraisal and optimization of the algorithm are possible, favoring rapid development and deployment. If the testing time is fast, real-time classification and online feedback are possible, favoring clinical implementation.

We first used 20–100% of the dataset (*n* = 2,881 functional primitives) in randomly selected 10% increments. At each increment, we measured (1) the time required to train the algorithm (training time), and (2) the time required for a trained algorithm to classify a primitive (testing time). At each 10% increment, the algorithms were trained *de novo* to avoid overfitting and to provide unbiased estimates.

Given the modest size of our dataset, we next used a simulated dataset that could be expected from a typical sample size of 50 subjects performing a variety of activities. The simulated dataset had 300,000 functional primitives with same proportion, mean, and variance as our original dataset. We used 25–100% of the dataset in randomly selected 25% increments. At each increment, we measured the training and testing times, training the algorithm *de novo* as above. Of note, the simulated dataset was used only to generate training and testing times, and was not used for classification performance assessments.

##### Tuning requirements of the algorithms

We also assessed the algorithm's need for tuning, the adjustment of algorithm parameters to maximize classification performance. A high tuning requirement requires the extensive analysis of the algorithm to identify its optimal parameters, potentially limiting implementation in settings that lack domain expertise. Of note, tuning requirements were only used to index complexity, but we did not tune the algorithms themselves in the assessment of classification performance.

We operationalized the algorithms' tuning requirements as the number of parameters that can be adjusted. We also qualitatively classified the level of domain knowledge required to implement and tune the algorithms. Based on typical US educational programs, “low” domain expertise indicates a basic knowledge of statistics, “medium” indicates undergraduate-level knowledge of machine learning, and “high” indicates graduate-level knowledge of machine learning.

#### Optimal Sensor Characteristics

We then focused on the hardware side, seeking the best balance between ease of motion capture and high classification performance. We first considered the use of IMUs compared to accelerometers alone. IMUs are a combination of sensors, including accelerometers, gyroscopes, and magnetometers. Many IMU hardware-software systems generate 3D linear accelerations, 3D angular velocities, 3D magnetic heading, and 4D quaternions, resulting in 10 data dimensions per sensor. We used accelerations, angular velocities, and quaternions for derivation of statistical features (section Methods), as these data types have been used previously for human activity recognition (20, 28, 29). In contrast, 3D accelerometers generate only 3D linear accelerations, resulting in 3 data dimensions per sensor.

While IMUs are data-rich, they are challenged by electromagnetic drift. Magnetic environments lead to potentially inaccurate gyroscopic measurements and therefore necessitate frequent recalibration. While accelerometers are data-sparse, they are largely unaffected by a magnetic environment.

Another practical consideration for sensor choice is system expense. IMU systems can cost thousands of dollars (11) whereas accelerometry systems cost in the hundreds (30). It is possible that cost and set-up time could be optimized by reducing the number of sensors or by using accelerometers alone. Although simplified and less expensive motion capture would favor clinical implementation, it may come at the cost of reduced classification performance.

In this analysis, we subsampled data from the IMUs to extract accelerometry data, ensuring that comparisons were based on identical sensor locations and primitive motions. LDA was trained and tested on the separate datasets to read out effects on classification performance.

##### Optimal sensor number and configuration for classification

We first evaluated how the number of sensors and their location on the body affects classification performance. We used exhaustive search to systematically test all possible sensor configurations (31). This approach provides an unbiased appraisal of all sensor combinations for each incremental reduction in sensor number.

##### Optimal sensor type for classification

We also evaluated how sensor type affected classification performance. We compared classification accuracies using IMU data vs. accelerometry-only data. This allowed us to determine whether accelerometers, with their reduced dimensionality, could enable sufficient accuracy to warrant their use in lieu of IMUs.

## Results

### Classification Performance of Algorithms

We first determined the classification performance of multiple ML algorithms using PPVs (Table 2). LDA and SVM had high classification performance for all functional primitives (overall PPV 92.5 and 92%, respectively). KNN had intermediate performance (PPV 87.5%) and NBC had the lowest performance (PPV 80.2%), particularly for *reaches* (PPV 77%) and *transports* (PPV 71%). In the leave-one-subject-out analysis, which addressed the possibility of within-subject dependencies, similar overall classification performances were identified (PPVs of 89% for LDA, 90% for SVM, 83% for KNN, and 75% for NBC).

**Table 2:** Classification performance of machine learning algorithms for functional primitives.

**Algorithm**	**PPVs for functional primitives**				**Overall PPV**
	**Reach**	**Transport**	**Reposition**	**Idle**	
LDA	93 ± 1.47%	91 ± 1.65%	93 ± 1.47%	92 ± 1.56%	92.5 ± 1.52%
NBC	77 ± 2.42%	71 ± 2.61%	83 ± 2.16%	85 ± 2.06%	80.2 ± 2.30%
SVM	92 ± 1.56%	90 ± 1.73%	92 ± 1.56%	93 ± 1.47%	92 ± 1.56%
KNN	86 ± 2.00%	87 ± 1.94%	85 ± 2.06%	89 ± 1.80%	87.5 ± 1.90%

*Positive predictive values (PPV) with associated 95% confidence intervals are shown. PPV reflects how often a primitive was actually made when the algorithm identified it as such, was calculated for the primitives of reach, transport, reposition, and idle. Primitive-level PPVs were computed in one-vs.-all analysis (e.g., reach vs. transport + reposition + idle combined). The overall PPV was assessed by combining data for all primitives and tallying all true and false positives. Overall classification performance was highest for linear discriminant analysis (LDA) and support vector machine (SVM), moderately high for k-nearest neighbors (KNN), and lowest for Naïve Bayes classifier (NBC)*.

**Figure 2:**
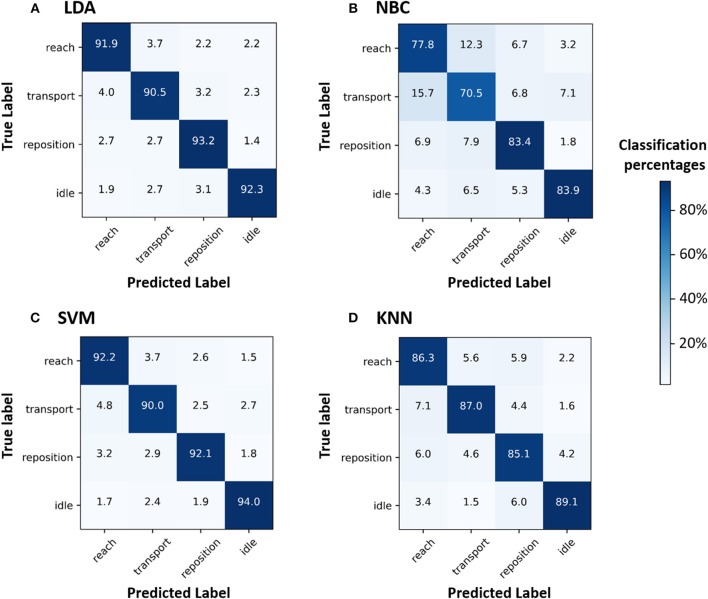
Confusion matrices (CMs) for the ML algorithms **(A)** LDA, **(B)** NBC, **(C)** SVM, and **(D)** KNN. CMs help visualize where ML algorithms are performing well (successful classification) or becoming confused (misclassification). Numbers in the diagonal cells represent the percentage of samples belonging to a class that were correctly classified by the ML algorithm. Numbers in the non-diagonal cells represent the percentage of samples belonging to a class (e.g., *reach*) that were classified by the ML algorithm as another class (e.g., *transport*). CMs with high values for diagonal cells indicate accurate classifications. In contrast, CMs with high values for non-diagonal cells indicate high misclassifications. The color bar indicates the percentage of primitives from a given class that has been classified as the same class (diagonal cells) or another class (non-diagonal cells). LDA and SVM accurately classified all four primitives, closely followed by KNN. NBC performed inadequately and became confused between *reaches* and *transports*.

We then inspected the confusion matrices of the algorithms, which enables us to identify primitive-level classification. We found that LDA and SVM had high classification success for all four primitives (diagonal cells in the confusion matrices: 90.0–94.0%) and had few misclassifications (non-diagonal cells: 1.4–4.8%) (Figure 2). KNN had moderate success in its classification of the four primitives (diagonal cells: 85.1–89.1%), and misclassified 7.1% of the *transports* as *reaches*. NBC had moderate success in its classification of *repositions* and *idles* (83.4 and 83.9%, respectively) and inadequately classified *transports* and *reaches* (70.5 and 77.8%, respectively). NBC misclassified 12.3% of the *reaches* as *transports* and 15.7% of the *transports* as *reaches*.

**Figure 3:**
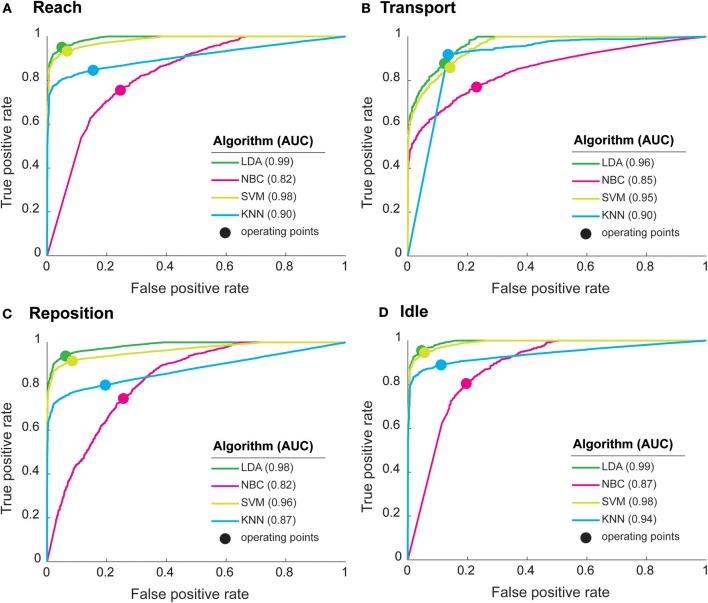
Performance characteristics of machine learning algorithms for **(A)** Reach, **(B)** Transport, **(C)** Reposition, and **(D)** Idle. Receiver operating characteristic (ROC) curves show the trade-off between true positive rate (or sensitivity) and false positive rate (1-specificity). Curves closer to the top-left corner indicate a better classification performance. The optimal operating point for each algorithm (solid circles), reflect the best tradeoff between sensitivity and specificity for an algorithm. The area under the curve (AUC), a measure of classification performance, is shown in parenthesis for each algorithm. AUC = 1 represents perfect classification. LDA had the highest AUCs followed closely by SVM, indicating high classification performances. NBC had consistently the lowest AUCs, indicating the weakest classification performance.

To further characterize classification performance, we generated ROC curves for each functional primitive (Figure 3). All algorithms detected *idle* with high accuracy (AUC > 0.87). For the other primitives, LDA and SVM had AUCs 0.95–0.99, indicating very high classification performance. KNN also had high classification performance for *reach* (AUC 0.94) and *transport* (AUC 0.90) and intermediate classification performance for *reposition* (AUC 0.87). In contrast, NBC had the lowest classification performance on the remaining primitives (AUC 0.80–0.85). We also identified the optimal operating point, indicating the best tradeoff between sensitivity and specificity, for each algorithm (Figure 3). At their respective optimal operating points, LDA and SVM achieved high sensitivities (0.83–0.95) and specificities (0.83–0.95) for all primitives. KNN achieved a high sensitivity (0.91) and specificity (0.86) for *transport*, but had moderate sensitivities (0.80–0.88) and specificities (0.79–0.86) for other primitives. NBC had the lowest sensitivities (0.74–0.81) and specificities (0.74–0.79) for all primitives. In sum, these findings indicate that LDA and SVM have the highest classification performance of the algorithms tested.

### Training and Testing Times of the Algorithms

**Figure 4:**
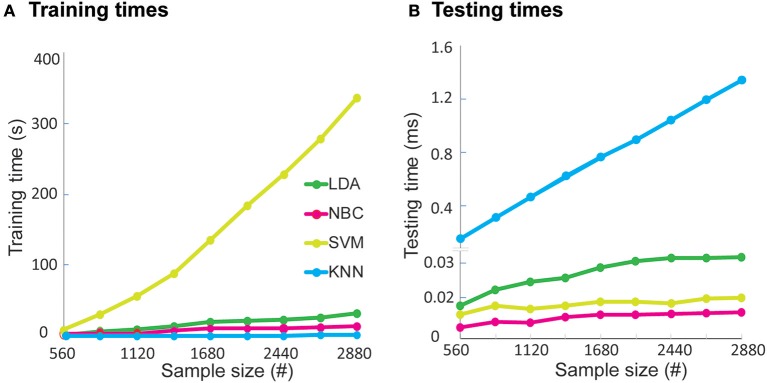
Algorithm **(A)** training times and **(B)** testing times on sample dataset. The dataset is comprised of 2,880 primitives. We computed times to train and test each algorithm on 20–100% of the dataset in increments of 10%. To avoid overfitting and compute an unbiased estimate of training and testing times, ML algorithms were trained and tested *de novo* with each incremental increase. For training with the complete sample dataset, SVM required the most time (336 s) while the other algorithms finished training rapidly (<30 s). For testing, KNN required the most time (1.5 ms), while the other algorithms finished testing rapidly (<0.03 ms). Please note break in the y-axis to highlight the difference in the algorithm testing times.

We next evaluated the pragmatic aspects of implementing the algorithm to gauge real-world applicability. We first calculated the time required to train and test the algorithm on increasing quantities of data (Figure 4) from our dataset of 2,880 functional primitives. In terms of training times, NBC and LDA were on the order of seconds (12 and 26 s, respectively), with training times growing linearly with increasing data quantity. SVM was on the order of minutes (5.6 min), with training times growing quadratically with increasing data quantity. KNN required no time to train as an inherent property of the model. In terms of testing, LDA, NBC, and SVM required sub-millisecond times (~0.03 ms), whereas KNN required the longest time (1.5 ms) with testing times growing linearly with increasing dataset size.

**Figure 5:**
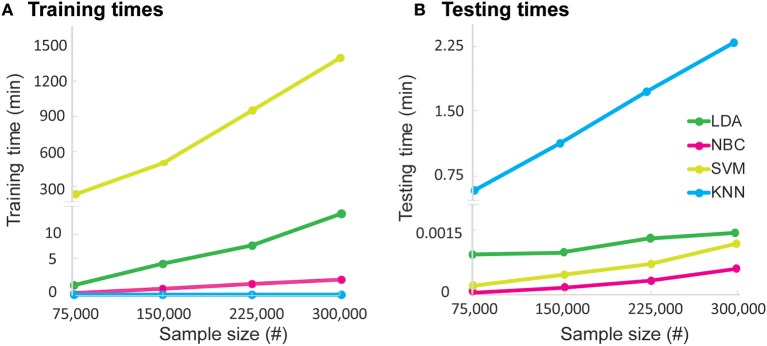
Algorithm **(A)** training times and **(B)** testing times on real world-sized dataset. The dataset is comprised of 300,000 simulated primitives. We evaluated training and testing times required by each algorithm for quartile increases in dataset size. Please note the break in the y-axes to highlight differences in training and testing times. To avoid overfitting and compute unbiased estimates, the algorithms were trained and tested *de novo* at each quartile. For training with the entire dataset, SVM required the most time (1,380 min) while the other algorithms required less time (LDA: 13 min; NBC: 2.5 min; KNN: 0 min, as per model property). For testing, KNN required the most time (2.3 min). The remainder of algorithms (LDA, NBC, and SVM) needed a testing time of <0.09 ms, which grew marginally with increasing sample sizes.

To investigate the real-world ramifications of training and testing requirements, we generated a dataset with 300,000 functional primitives (Figure 5). Training times became prohibitively long for SVM (up to 23 h) but were manageable for the other algorithms (up to 13 min). Testing time was relatively high for KNN (up to 2.3 min), whereas LDA, NBC, and SVM required nominal testing times (<0.03 ms). Given their consistently low training and testing times, LDA and NBC have the best practical performance of the algorithms tested.

### Tuning Requirements of the Algorithms

To gauge the difficulty of algorithm implementation, we characterized their tuning requirements (Table 3). NBC has the lowest number of parameters (1) and requires a low amount of domain knowledge in machine learning to optimize it. KNN has a moderate number of parameters (5), but their optimization is reasonably intuitive and requires a low level of domain knowledge. LDA has fewer parameters (3), but they require a medium level of domain knowledge. SVM has many parameters (9) and requires a high level of domain knowledge to build an accurate and efficient model. In sum, these findings indicate that NBC and KNN are the easiest to implement, and LDA implementation requires a modestly higher skillset.

**Table 3:** Complexity of algorithm implementation.

**Algorithm**	**# Tuning parameters**	**Tuning parameters**	**Level of domain knowledge**
LDA	3	Prior probability, regularization term, optimizer	Medium
NBC	1	selection of prior distribution	Low
SVM	9	Kernel function, kernel parameters (scale, offset), regularization term, # of iterations, Nu, prior probability, convergence parameter, optimizer	High
KNN	5	# of neighbors (K), distance metric, search algorithm, tie breaker, weighing criterion	Low

*Algorithm parameter tuning is necessary to achieve optimal classification performance. Shown are algorithm tuning characteristics, as indicated by number and specifics of the tuning parameters. Also shown is a graded estimate of the level of domain knowledge required to tune these parameters. NBC is considered the simplest to tune while SVM is the most difficult. LDA has a handful of parameters that require medium domain knowledge to negotiate. KNN has a moderate number of parameters that are intuitive to tune and require little domain knowledge. Level of domain knowledge: low, basic knowledge of statistics; medium, undergraduate-level knowledge of ML; high, graduate-level knowledge of ML*.

### Optimal Sensor Characteristics

#### Optimal Sensor Number and Configuration

**Figure 6:**
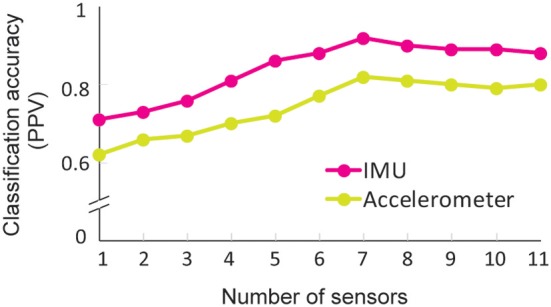
Classification performance for full and reduced sensor counts. Performance was computed using LDA and data from with progressively reduced sensor counts. Seven sensors (pelvis, sternum, head, and the active shoulder, upper arm, forearm, and hand) gave the best classification performance, with a performance drop-off at more or fewer sensors. IMU data consistently supported higher classification than accelerometery data, achieving PPV 92.5 vs. 82% at the seven sensors.

To evaluate the effect of the sensor number and configuration on classification performance, we used an exhaustive search process, which evaluated all combinations of sensor number and location. We note that exhaustive search arrived at the same optimal configurations for IMUs as for accelerometers. Seven sensors on the head, sternum, pelvis, and UE of the active side resulted in the highest classification performance (IMU PPV 92.5%; accelerometer PPV 84%). In comparison, when exhaustive search progressively added sensors to the non-active forearm, then hand, then upper arm, then scapula, classification performance worsened (IMU PPV 88%; accelerometer PPV 80%) (Figure 6). When exhaustive search progressively removed sensors on the trunk and then head, performance also worsened. Subsequent removal of sensors from the scapula, then arm, and then hand further worsened performance, arriving at PPVs of 71 and 62% for IMUs and accelerometers, respectively, for the remaining forearm sensor.

#### Optimal Sensor Type for Classification

To finish, we evaluated classification performance using IMU vs. accelerometry data only. Classification performance using accelerometry data was consistently lower than for IMU data for all sensor configurations (Figure 6; Table 4). Classification performance with accelerometers was lower especially for *reaches* (PPV 77 vs. 93%; Table 4), which include different arm configurations to grasp the objects (e.g., supinating to side-grasp the aluminum can vs. pronating to overhand grasp the toilet paper roll). These findings indicate that IMU data enable a superior level of classification, particularly with more variable motions involving forearm rotations.

**Table 4:** Primitive-level classification using IMU or accelerometer data.

**Primitives**	**Classification performance (PPV)**	
	**IMU**	**Accelerometer**
Reach	93%	77%
Transport	91%	80%
Reposition	93%	82%
Idle	92%	88%
Average	92.5%	82%

*Classification performance is shown using the 7-sensor configuration (pelvis, sternum, head, and the active shoulder, upper arm, forearm, and hand). Accelerometers provided systematically poorer classification performance compared to IMUs across all primitives. Classification performance using accelerometry data was particularly low for reach (PPV 77%) and relatively higher for idle (PPV 88%)*.

## Discussion

The combination of wearable sensors and machine learning offers exciting opportunities in numerous applications, including human activity recognition (1–3) and assessment of impaired motion (5, 8, 9). We recently proposed an approach that uses wearable sensors and ML algorithms to classify functional primitives, which could be summed to quantify rehabilitation dose. In this study, we aimed to address limitations in this previous work, including a modest computational performance, high computational complexity, and hardware drawbacks. We present our analyses as a primer for considering software and hardware variables in the capture and classification of motion data. We sought to identify—from both performance and practical standpoints—the best machine learning algorithm, sensor configuration, and sensor type to classify primitives in stroke patients.

Among the ML algorithms, LDA represented the best balance of classification performance and pragmatic implementation. Among sensor configurations, seven sensors on the paretic arm and trunk enabled better classification performance than more or fewer sensors on the body. Among sensor types, IMU data enabled better classification performance than accelerometers. To our knowledge, this is the first study to systematically outline the steps of identifying optimal ML algorithms, sensor configurations, and sensor types to automatically classify motion patterns of neurological patients.

### Optimal Performer in Classification

Evaluating the ability of the ML algorithms to classifying functional primitives, we found that LDA and SVM had the highest classification performance. LDA performs well because it aims to reduce dimensionality while preserving as much discriminatory information as possible. This approach leads to tight clusters and high separation between the classes (32). SVM performs well because it projects training data to a high-dimensional space. This approach leads to maximal separation between classes that may not be possible in the original feature space (33). Overall, LDA aims to find commonalities within and differences between data classes, whereas SVM aims to find a classification boundary that is furthest from the data classes. Importantly, these algorithms maximize rigor in the training phase by being less susceptible to noisy or outlier data (34, 35). LDA accomplishes this by using the clusters' centers and ignoring outlier samples to classify (34), while SVM uses the most closely spaced data (i.e., the most difficult to discriminate) to define class boundaries (35). It is worth noting that LDA assumes that the underlying classes are normally distributed (unimodal Gaussians) with the same covariance matrix (32). If real-world motion data are significantly non-Gaussian, LDA may not capture the complex data structures required for accurate classification. In this case, classification performance can be tuned by allowing the covariance matrices among classes to vary, resulting in a regularized discriminant analysis (36).

By comparison, KNN showed a marginally lower classification performance, likely due to its susceptibility to noise (37). KNN relies on the assumption that samples from the same class exist in close proximity. Given a new sample, KNN assigns it to the class with the majority of closest neighbors (38). In our current setup of KNN, all nearest sample points are given the same weighting. Therefore, when assigning a class label, a noisy sample will be weighted the same as other statistically important samples. KNN classification performance can be tuned by choosing an appropriate weighting metric (e.g., inverse squared weighing) (39), which ensures that samples closer to the test sample contribute more to classifying it. Performance may also be tuned by using mutual nearest neighbors, where noisy samples are detected using pseudo-neighbors (neighbors of neighbors) and are assigned lower weights (40).

Finally, NBC had the lowest performance compared to other algorithms. NBC uses Bayes' rule and prior information to classify a new sample, using the posterior probability of it belonging to a class (41). Its lower performance may be attributed to its underlying assumption of conditional independence between data features (42). This assumption is violated for data streams that are correlated, such as data from adjacent sensors on the body, like the hand and wrist. The performance of NBC could be improved by applying principal components analysis to the dataset as a pre-processing step, and then training the NBC (43).

Comparing these results with our prior work (10), we found that the four algorithms outperformed the hidden Markov model-logistic regression (HMM-LR) classifier for identifying the functional primitives in stroke patients. The improved performance may be due in part to differences in the training datasets. Our previous study trained the algorithm on healthy control data and tested on stroke patient data to examine the generalizability of the model. It is conceivable that if the HMM-LR classifier been trained and tested in stroke patients only, its performance would have been higher. To enable a fair comparison of classification accuracy with our previous study (10), we used the same statistical features. We did not perform feature selection, which is the process of selecting the most informative statistical features to obtain a subset of the original feature set (44). Feature selection reduces the data dimensionality, which in turn reduces overfitting by ML algorithms, lowers training time, and increases classification accuracy (45, 46). Given these benefits, a clinician or researcher investigating a new problem of interest is encouraged to consider feature selection on their dataset.

We also generated confusion matrices to further characterize where ML algorithms are performing well (successful classification) vs. becoming confused (misclassification). In our analysis, we observed that NBC had limited success in differentiating between *reaches* and *transports*, functional primitives whose motion patterns are quite similar (12). The main difference between these primitives are grasp-related wrist motions (wrist extension and supination) occurring during *reach* (12). To accurately disambiguate these primitives with a lower-performing algorithm, we would emphasize [i.e., assign higher weights to (47)] data from the distal UE segment. This analysis shows how confusion matrices can help an investigator target and address areas for improvement in ML classification.

### Optimal Performer in Practicality

We also determined the most pragmatic algorithms with respect to their training and testing times and their tuning requirements. In terms of training times, KNN did not have any computational overhead. This is expected, since KNN requires no training and shifts its computations to the testing phase. Training times for LDA and NBC grew gradually with dataset size, but took at most minutes with a real world-sized dataset. LDA had lower training times than NBC on a smaller dataset, but required more training time as the dataset increased. This is explained by the scatter matrix computations and optimization of LDA, which become computationally expensive as the dataset size increases (22). By contrast, SVM training time increased quadratically with dataset size, because finding an optimal hyperplane between classes entails solving a quadratic programming problem (23). Complex algorithms such as SVM thus require more processing time for large datasets, which limits real-world application. For example, for a modestly sized study, training times for SVM may be on the order of days. This lag would be prohibitive for rapid tuning, significantly delaying algorithm optimizations. Conversely, performance of LDA and NBC could be rapidly appraised after training, alerting an investigator to further tune the algorithm or to move on from it.

In terms of testing times, SVM, LDA, and NBC required sub-milliseconds to classify functional primitives, whereas KNN took seconds-minutes and testing times grew linearly with dataset size. This can be explained by the exhaustive and computationally expensive search performed by KNN (48). During testing, the KNN algorithm searches for the k nearest neighbors that have similar data characteristics as the test sample. With increasing samples and dimensionality of the data, the search broadens and takes more time. If an investigator wishes to classify primitives offline, KNN testing times may be acceptable. For applications requiring near- or real-time classification (e.g., for online feedback), the other algorithms should be considered instead. Alternatively, the computational complexity of KNN can be reduced by selecting an efficient search algorithm (e.g., KD tree) (49), which limits the search space during testing.

In terms of ease of tuning to increase classification performance and reduce training/testing time, we determined that NBC had the lowest parameter complexity and requirement for domain knowledge in machine learning. KNN has a moderate number of tuning parameters, but they are relatively straightforward to understand and address. LDA has fewer tuning parameters than KNN, but moderate domain knowledge is required to select the amount of regularization allowing the covariance among classes to vary (36). SVM requires the highest amount of parameter tuning, and necessitates a deep understanding of statistics, optimization, probability theory, and machine learning (50). This level of domain knowledge is prohibitive for SVM use in an unsupported research setting.

Weighing classification performance and pragmatic implementation, we judged LDA to be the best choice for our application. Investigators will similarly need to weigh their performance goals, time resources, and available level of expertise for ML implementation in their own motion classification questions.

### Optimal IMU Configuration

On the hardware side, we determined the optimal sensor location and configuration to facilitate data capture while maintaining high classification performance. Seven sensors (not more or fewer) enabled optimal classification performance, and the best sensor configuration was placed on the active limb and trunk. This result is expected, given that the participants performed a unimanual task. Interestingly, accuracy worsened with more sensors, likely because of the increased dimensionality of the dataset. This may cause the ML algorithm to overfit the training data, resulting in lower classification performance during the testing phase (51). Finally, we found that if only one sensor was available, the forearm location was the most informative, although classification performance was modest. This location is nonetheless appealing, given recent advances in smartwatches capable of capturing motion.

### Optimal Data Characteristics

Finally, we determined the sensor type that led to the highest classification performance. Accelerometry data consistently generated lower accuracies than IMU data, likely due to its fewer dimensions. Although IMUs enable higher classification performance than accelerometers, they also have some drawbacks: a higher risk of electromagnetic drift leading to inaccurate data estimates and the need for more frequent recalibrations, a higher consumption of energy (52), and a higher cost (11). Thus, there is a tradeoff between robust motion capture and practical motion capture. We believe that the benefits of richer data and better classification of IMUs outweigh their practical limitations. However, there currently exist no benchmarks for the level of classification accuracy needed to justify clinical implementation. If these accuracy benchmarks are lower than those achieved by IMUs, and if investigators are constrained by financial resources or the magnetic noisiness of an environment, accelerometers could be appropriate.

## Machine Learning for Other Clinical Application

Machine learning is a potentially powerful tool for myriad clinical applications, by virtue of its automatic recognition of categories of interest. Investigators may seek to use ML diagnostically, as with classification of disease states or severity. For example, investigators can grade Parkinson's disease based on classes of motion defined by severity of tremor, bradykinesia, and dyskinesia (5, 6, 53). Investigators may also seek to use ML therapeutically, as with performance assessments. For example, investigators can identify and count classes of behavioral output in training contexts, such as the number of words uttered during speech therapy (54) or the number of functional primitives performed during occupational therapy (12).

Notably, ML models will differ depending on the goal. There exists no universal classifier; in other words, no single model can classify all categories that could be of interest to researchers and clinicians. For a ML algorithm to identify categories of interest, it has to be trained to recognize the data features that correspond to these categories. This means that the appropriate subject groups, well-defined classes, and trained observers are needed to generate and label the data for subsequent algorithm training and testing.

## Limitations and Future Work

Our study has some limitations to be considered. The present work showcases the use of the ML- sensor approach, providing head-to-head comparisons between ML algorithms and sensor type. Importantly, we demonstrate its use for a particular clinical question; we did not build a universal system that applies to all neurological disorders. Also importantly, we did not identify the definitive approach for classifying functional primitives in all rehabilitating stroke patients, for two reasons.

First, our analysis was performed on a dataset of mild-to-moderately impaired stroke patients. This not only limits generalization to stroke patients with severe impairment, but also reduces the variability of the dataset upon which the algorithms were trained and tested. Off-the-shelf algorithms may be inadequate for more variably impaired subject populations. To handle variability in the data, advanced machine learning approaches such as deep learning will likely be needed for accurate classification. For these more complex datasets, collaboration with experts in machine learning, who can apply and refine deep learning architectures, is suggested.

Second, the activity used in this study was highly structured. The motion characteristics of the resulting primitives were thus more consistent and limited than would be found in a real-world rehabilitation setting. The training and testing of algorithms on data with more varied kinematics is still required, and is ongoing in our laboratory. We used this circumscribed and controlled dataset here so that we could focus our appraisal on the ML and sensor methodologies. More importantly, it allowed us to display the practical deliberations required in the development of a sensor-ML approach for motion classification.

## Conclusion

In summary, we present a primer that details how one can optimize both the software and hardware facets of motion capture. This work outlines computational and practical considerations for implementing a sensor-ML approach in quantitative research. Specific to our application, we demonstrate how to refine a strategy that builds toward the precise and pragmatic classification of functional primitives in stroke patients. We found that LDA had the best combination of classification performance and pragmatic performance. We also found that seven sensors on the paretic UE and trunk maximized classification performance, and that IMUs enabled superior classification compared to accelerometers.

## Data Availability

The dataset analyzed for the current study are available from the corresponding author on reasonable request. Code for data analysis has been made is available on github https://github.com/avinashparnandi/MLPrimer.

## Ethics Statement

The studies involving human participants were reviewed and approved by Institutional Review Board at Columbia University. The patients/participants provided their written informed consent to participate in this study.

## Author Contributions

AP analyzed and interpreted the data and wrote the paper. JU collected and labeled the data. DN created the activities battery and interpreted the data. HS created the activities battery, collected, labeled, interpreted the data and wrote the paper.

### Conflict of Interest Statement

The authors declare that the research was conducted in the absence of any commercial or financial relationships that could be construed as a potential conflict of interest.
